# Diplotyper: diplotype-based association analysis

**DOI:** 10.1186/1755-8794-6-S2-S5

**Published:** 2013-05-07

**Authors:** Sunshin Kim, KyungChae Park, Chol Shin, Nam H Cho, Jeong-Jae Ko, InSong Koh, KyuBum Kwack

**Affiliations:** 1 Department of Biomedical Science, College of Life Science, CHA University, Seongnam, Korea; 2 Department of Family Medicine, CHA Bundang Medical Center, CHA University, Seongnam, Korea; 3 Division of Pulmonary and Critical Care Medicine, Department of Internal Medicine, Korea University Ansan Hospital, Ansan, Korea; 4 Department of Preventive Medicine, Ajou University School of Medicine, Suwon, Korea; 5 Department of Physiology, College of Medicine, Hanyang University, Seoul, Korea

## Abstract

**Background:**

It was previously reported that an association analysis based on haplotype clusters increased power over single-locus tests, and that another association test based on diplotype trend regression analysis outperformed other, more common association approaches. We suggest a novel algorithm to combine haplotype cluster- and diplotype-based analyses.

**Methods:**

Diplotyper combines a novel algorithm designed to cluster haplotypes of interest from a given set of haplotypes with two existing tools: Haploview, for analyses of linkage disequilibrium blocks and haplotypes, and PLINK, to generate all possible diplotypes from given genotypes of samples and calculate linear or logistic regression. In addition, procedures for generating all possible diplotypes from the haplotype clusters and transforming these diplotypes into PLINK formats were implemented.

**Results:**

Diplotyper is a fully automated tool for performing association analysis based on diplotypes in a population. Diplotyper was tested through association analysis of hepatic lipase (*LIPC*) gene polymorphisms or diplotypes and levels of high-density lipoprotein (HDL) cholesterol.

**Conclusions:**

Diplotyper is useful for identifying more precise and distinct signals over single-locus tests.

## Background

Causal mutations for health conditions with genetic bases can be identified through finding associations with haplotypes, a form of correlation known as linkage disequilibrium (LD) [1]. Investigating significant haplotype structure has become a routine research activity. The Haploview tool provides computation of LD and population haplotype patterns from genotype data [2]. The PLINK tool set [3] accomplishes diverse functions including a module carrying out Expectation-Maximization (EM) algorithm [4]. PLINK focuses on fast calculations with large datasets. WHAP was developed to perform haplotype-based association analysis in population and family samples using single nucleotide polymorphism (SNP) data [5]. An additional software tool was elaborated for carrying out haplotype association analysis in unrelated individuals [6]. To provide a detailed genome structure, a recloning system [7] was developed to obtain the sequences of 20 haplotypes from a chimpanzee and a gorilla, across human leukocyte antigen (HLA) genes.

Meanwhile, rare haplotypes have been investigated to identify their roles in influencing disease susceptibility. Experimental data showed that two rare haplotypes of parathyroid hormone-related peptide receptor type 1 and vitamin D receptor genes, with frequencies of 1.1% and 2.9%, respectively, were significantly associated with osteoporosis phenotypes (*P *= 4.2 × 10^-6 ^and *P *= 1.6 × 10^-4^, respectively) [8]. Recently, haplotypes in the regulatory regions of the HLA-G gene were examined to recognize possible associations with the implantation outcome in couples undergoing assisted reproduction treatments (ART). The results revealed a complete absence of some haplotypes in couples undergoing ART [9].

Notably, Durrant et al. proposed a novel approach to investigate associations between diseases and haplotype clusters in a logistic regression framework through cladistic analysis of SNP haplotypes. Substantial increases in power over single-locus tests were demonstrated by the simulation study. Their empirical data showed that a haplotype cluster that consisted of two haplotypes had the strongest effect on Cystic Fibrosis (OR = 96.8) [10]. Luo et al. used a novel analysis, diplotype trend regression (DTR) analysis, to investigate associations between certain diplotypes of alcohol dehydrogenase and aldehyde dehydrogenase genes, and alcohol dependence. They demonstrated that DTR outperformed other conventional association methods [11]. Both articles indicated that our new algorithm might provide a synergistic effect through combining analyses based on both haplotype clusters and diplotypes.

Here, we propose a novel method to investigate associations between diplotypes and diseases. We define a haplotype cluster as a set of haplotypes. We also define a diplotype as a haplotype cluster pair, the definition of which is extended from a haplotype pair. The first step of our method uses the Haploview tool to generate all possible haplotypes. Second, all possible haplotype pairs (diplotypes) from SNP genotypes of all samples are generated by PLINK. Third, all possible haplotype clusters are generated by our clustering algorithm from the haplotypes produced in the first step. Fourth, the patterns of all possible diplotypes are generated from those haplotype clusters. Fifth, to calculate regression by PLINK, the diplotypes of the samples are transformed into AA, AB, or BB formats according to the diplotype patterns produced in the fourth step. Finally, PLINK was used with a regression model to obtain the association results. All of these procedures are performed automatically by the software we developed, named Diplotyper, which was implemented in Python 2.7.

We applied this method to an association study between high-density lipoprotein cholesterol (HDL-C) and the hepatic lipase (HL) gene. HL is involved in lipoprotein metabolism through its bridging function, which facilitates the interaction between lipoproteins and lipoprotein receptors, and its activity plays an important role in plasma lipoprotein metabolism and the atherosclerotic process [12]. HL plays an important role in both reverse cholesterol transport and non-cholesterol-dependent mechanisms involved with HDL [13,14]. Changes in HL activity can be associated with alterations in lipoprotein composition, which may contribute to the development of atherosclerosis [12,14]. Low HDL-C levels are risk factors for coronary heart and cardiovascular diseases [15,16]. Extensive research has provided evidence that increasing HDL-C levels can reduce the risk of cardiovascular disease [17-20]. The risk of developing coronary heart disease can be determined by the levels of HDL-C [21]. In particular, a recent report, based on the Korea National Health and Nutrition Examination Surveys I, II, III and IV, shows a growing prevalence of dyslipidemia and hypertriglyceridemia in Korea during the last decade [22].

*LIPC *encodes hepatic triglyceride lipase, which is expressed mainly in the liver and is located on 15q21-q23, where it spans 171 kb and comprises nine exons and eight introns. Two SNPs [-514C > T (rs1800588) and -250G > A (rs2070895)] in the promoter region are in almost perfect LD (R^2 ^= 0.97) [23,24] and both the promoter SNP (rs1800588) and the intronic SNP (rs261332) has strong LD (R^2 ^= 0.92) in HapMap CEU (Caucasians of European descent from Utah) database [25,26]. The intronic SNPs rs261332 [26] and rs11858164 [27], and the promoter SNPs rs1532085 [28] and rs10468017 [29], were associated with HDL-C levels in genome-wide association studies. The promoter SNP rs1800588 showed an increase in HDL-C of 0.04 mmol/l in the CT group and 0.09 mmol/l in the TT group, compared with the CC carriers [30]. The promoter SNP rs2070895 showed a highly significant association with a 0.057 mmol/l increase in HDL-C per A allele (*P *= 8 × 10^-10^) [24]. We investigated possible associations between *LIPC *SNPs or diplotypes and HDL-C levels in a Korean population consisting of 7,536 individuals.

## Methods

### Algorithm

A haplotype cluster is defined as a set of haplotypes. A diplotype is defined as a haplotype cluster pair, the definition of which is extended from a haplotype pair. The diplotype is a homozygous diplotype or a heterozygous diplotype. LD blocks based on Gabriel et al. [31] or the Four Gamete algorithm [32] or the Solid Spine method (2), along with the haplotypes generated by EM algorithm, were used.

The first step produces LD blocks and any haplotypes for these LD blocks adjusting a threshold frequency, using Haploview tool.

The second step produces all possible haplotype pairs from SNP genotypes of samples employing PLINK, which implements the EM algorithm.

The third step starts with a set of haplotypes, *H *= {*H_1_, ..., H_n_*}, which is obtained in the first step. Another set, *HS *= {{*H_1_*}, ..., {*H_n_*}}, consists of subsets with a single element of *H*. Repeat frequency is initialized to 0. The procedure in this step is as follows.

**Procedure**: If the length of the *HS *subset is greater than the repeat frequency and none of the elements of the *HS *subset are equal to any element of *H*, and the last element of *HS *subset is less than each element of *H*, each element of *H *is added to *HS *subset. In this way, *HS *is updated and the repeat frequency increases by one. The procedure is repeated until the repeat frequency plus one is equal to the total number of *H *elements. Table 1 represents an example of the input and output data (haplotype clusters) in the case of *H *= {*A, B, C, D*}.

**Table 1:** Haplotype clusters.

Input	*H *= {*A, B, C, D*}, *HS *= {{*A*}, {*B*}, {*C*}, {*D*}}*, repeat frequency = 0*
Output	*HS *= {{*A*}, {*B*}, {*C*}, {*D*}, {*A, B*}, {*A, C*}, {*A, D*}, {*B, C*}, {*B, D*}, {*C, D*}, {*A, B, C*}, {*A, B, D*}, {*A, C, D*}, {*B, C, D*}, {*A, B, C, D*}}

Example of the input and output data (haplotype clusters) when *H *= {*A, B, C*, *D*}.

The fourth step produces the patterns of all possible diplotypes from the results of the third step. Table 2 represents an example of the output data in this step. The "*" in "{*A*}/*" indicates a set of all haplotypes except the "*A*" haplotype. Therefore, the set includes not only the haplotypes with frequencies greater than a threshold, but also those with frequencies below a threshold.

**Table 2:** Patterns of all possible diplotypes.

Output	{*A*}/*, {*B*}/*, {*C*}/*, {*D*}/*, {*A, B*}/*, {*A, C*}/*, {*A, D*}/*, {*B, C*}/*, {*B, D*}/*, {*C, D*}/*, {*A, B, C*}/*, {*A, B, D*}/*, {*A, C, D*}/*, {*B, C, D*}/*, {*A, B, C, D*}/*
	
	{*A*}/{*B*}, {*A*}/{*C*}, {*A*}/{*D*}, {*B*}/{*C*}, {*B*}/{*D*}, {*C*}/{*D*}, {*A*}/{*B, C*}, {*A*}/{*B, D*}, {*A*}/{*C, D*}, {*B*}/{*A, C*}, {*B*}/{*A, D*}, {*B*}/{*C, D*}, {*C*}/{*A, B*}, {*C*}/{*A, D*}, {*C*}/{*B, D*}, {*D*}/{*A, B*}, {*D*}/{*A, C*}, {*D*}/{*B, C*}, {*A*}/{*B, C, D*}, {*B*}/{*A, C, D*}, {*C*}/{*A, B, D*}, {*D*}/{*A, B, C*}, {*A, B*}/{*C, D*}, {*A, C*}/{*B, D*}, {*A, D*}/{*B, C*}

Example of the output (diplotypes) produced from the results (input) of the third step.

In the final step, the diplotypes from samples are transformed into AA, AB, or BB formats according to the patterns of the diplotypes generated in the fourth step. For example, the diplotypes, "{*A*}/*", consist of three possible diplotypes, "{*A*}{*A*}", "{*A*}*", or "**", just as the SNP alleles (A/B) comprise three genotypes (AA, AB, or BB). That is, input formats for PLINK can be made from the diplotypes of samples in a population. At this point, linear or logistic regression analysis can be carried out by PLINK.

### Software

Diplotyper is freely available on the website http://code.google.com/p/diplotyper/downloads/list.

### Subjects

The population data used in the present study was provided by the Korea Association Resource (KARE) project from the Korean Genome Epidemiology Study (KoGES), which is conducted by the Korean National Institute of Health (KNIH). A cross-sectional analysis of samples from urban and rural communities in Korea was conducted. The populations [33] were recruited from rural (Ansung) and urban (Ansan) communities in South Korea that were part of the KoGES, established in 2001. A total of 5,018 subjects from the Ansung community and 5,020 subjects from the Ansan community participated in the present study. The age of the participants ranged from 40 to 69 years. A total of 8842 subjects remained after selection of samples for quality control purposes [33]. However, an additional 1,306 subjects who were undergoing treatment for hypertension, diabetes, myocardial infarction or hyperlipidemia were excluded from the study since therapy for these conditions could change HDL-C levels. A detailed list of the characteristics of the study participants is shown in Table 3.

**Table 3:** Features of the study subjects.

	KARE project		
	
	Ansung (*n *= 3434)	Ansan (*n *= 4102)	Total (*n *= 7536)
Age	54.8 ± 8.8	48.2 ± 7.4	51.2 ± 8.7
Gender			
Male (%)	1534 (44.7)	2104 (51.3)	3638 (48.3)
Female (%)	1900 (55.3)	1998 (48.7)	3898 (51.7)
BMI (kg/m^2^)	24.2 ± 3.2	24.5 ± 2.9	24.4 ± 3.1
HDL-C (mmol/l)	1.2 ± 0.3	1.2 ± 0.3	1.2 ± 0.1

Features of the study subjects included in the KARE project.

Data are represented as mean ± SD, or as the observed number followed by its corresponding percentage.

BMI, body mass index; HDL, high-density lipoprotein.

### Genotyping

All *LIPC *38 intronic SNPs available to the research community through the KARE project from KoGES were analyzed. The study protocol was approved by the Institutional Review Board of KNIH. The genotyping of the samples from the Ansung and Ansan cohorts was performed using the Affymetrix Genome-Wide Human SNP Array 5.0 (Affymetrix Inc., Santa Clara, CA, USA). The Bayesian Robust Linear Model with the Mahalanobis distance genotype-calling algorithm was used with the Affymetrix SNP array 5.0. The SNPs were filtered if any of the following criteria were met: i) a call rate lower than 95%, ii) a minor allele frequency (MAF) lower than 0.05, or iii) a significant deviation from the Hardy-Weinberg equilibrium (HWE) lower than 1 × 10^-6^.

### Biochemical measures

Biochemical data from the KoGES were obtained through the KARE project. Blood samples were collected from the participants after at least eight hours of overnight fasting. HDL-C concentrations were measured with the Advia 1650 analyzer (Siemens, Tarrytown, NY, USA) for the Ansung and Ansan cohorts.

### Statistical analyses

To examine possible associations between *LIPC *SNPs or diplotypes and HDL-C levels, linear regression analyses were conducted with adjustments for area, age, gender and body mass index (BMI). An additive model was assumed for this study. Log transformation was applied to HDL-C values to normalize their distribution. Associations were evaluated as significant at a level of 0.05 after Bonferroni correction for multiple testing. This conservative (Bonferroni) adjustment required *P *values of 2.23 × 10^-4 ^in HDL-C before correction, since testing of 224 independent hypotheses (38 single-locus and 186 diplotype tests) for HDL-C was assumed. To determine the effects of SNPs and diplotypes, untransformed HDL-C concentrations were used. Statistical analyses were performed using PLINK version 1.07 (http://pngu.mgh.harvard.edu/~purcell/plink) and Python (version 2.7.1; Python Software Foundation, Wolfeboro Falls, NH).

## Results

**Figure 1:**
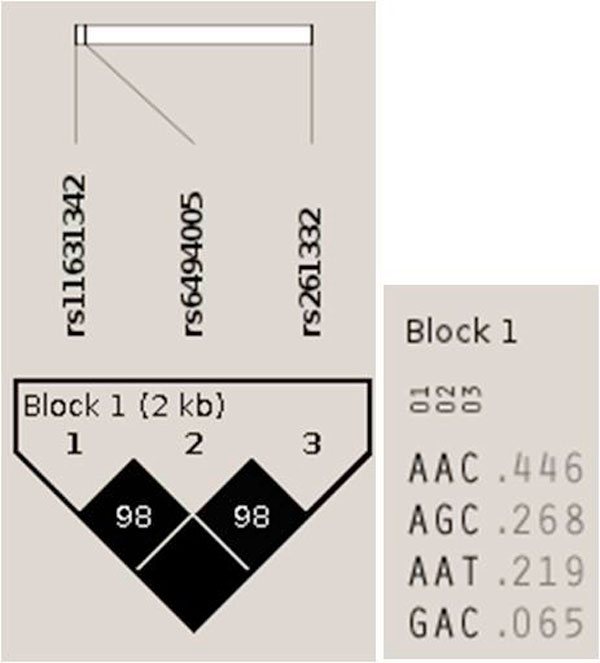
**LD plot of block 1**. The LD plot represents the pair-wise D' and haplotype frequency.

The total number of tests of association in the additive model were 224 for HDL-C (data not shown). As shown in Table 4, out of all *LIPC *38 intronic SNPs available to the research community through the KARE project, only three SNPs were selected, since these were the only ones with strong associations with HDL-C that were able to withstand Bonferroni correction (*P *< 2.23 × 10^-4^, see Methods), on the basis of their diplotypes as well as their SNP genotypes. The SNP rs261332 had the strongest association (*P *value = 3.03 × 10^-12^) with HDL-C levels. Figure 1 shows the LD block and the haplotypes for this block that exhibited frequencies of greater than the 1% threshold.

**Table 4:** Associations of single SNPs.

SNP	Minor; Major	Genotype frequency	Effect	Standard error	*P *value
rs11631342	G; A	29/923/6583	0.037	0.0083	5.66E-06
rs6494005	G; A	569/2891/4018	-0.023	0.0047	7.43E-07
rs261332	T; C	350/2617/4556	0.035	0.0049	3.03E-12

Associations of single SNPs with HDL-C levels.

Effect sizes (mmol/l) shown are β-coefficients and measured as additive effects, which correspond to the average change in HDL-C levels when one major allele is replaced with one minor allele.

**Figure 2:**
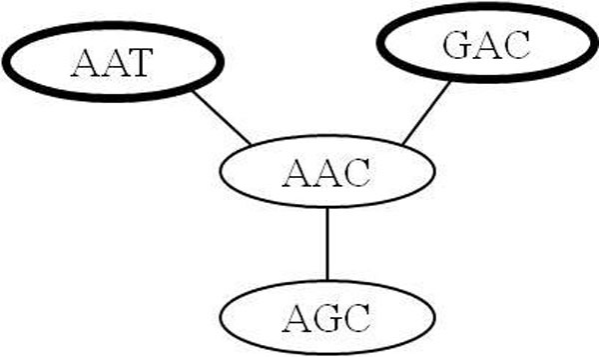
**Genealogical tree**. The thick and thin ellipses have higher and lower HDL-C levels, respectively.

Table 5 represents the results of the Diplotyper tool, which indicate very diverse associations between diplotypes and HDL-C levels. The top 10 results out of the 40 diplotypes (data not shown) in block 1, in terms of *P *value, are shown. The abbreviation b1 means block 1, H12 means the twelfth haplotype cluster, and OH12(*) represents all other haplotypes (including haplotypes below 1% frequency), except the H12 haplotype cluster; b1_H12 is a minor haplotype cluster and b1_OH12(*) a major haplotype cluster. The number of one homozygous diplotype (a b1_H12 pair) was 612, the number of a heterozygous diplotype (b1_H12 and b1_OH12(*)) was 3,064, and the number of another homozygous diplotype (a b1_OH12(*) pair) was 3,858. The strongest association with HDL-C levels showed a *P *value of 9.09 × 10^-19 ^and was much stronger than the *P *value of the single SNP rs261332. Figure 2 shows the genealogical tree visualized from HapStar tool [34]. The haplotypes (AAT or GAC) were associated with higher HDL-C levels and the haplotypes (AAC or AGC) were associated with lower HDL-C levels. The association of **A**A**C **/ AA**T **or **G**AC (different in bold) with HDL-C levels was statistically significant, but the association of A**A**C/A**G**C (different in bold) with HDL-C levels was not statistically significant in (Table 6, Figure 1, 2).

**Table 5:** Associations of diplotypes.

Diplotypes	Minor; Major	Diplotype frequency	Effect	Standard error	*P *value
b1_H12(AAT-or-GAC)/b1_OH12(*)	b1_H12; b1_OH12	612/3064/3858	0.041	0.0044	9.09E-19
b1_H6(AAC-or-AGC)/b1_OH6(*)	b1_OH6; b1_H6	617/3064/3853	0.040	0.0044	1.04E-18
b1_H6(AAC-or-AGC)/b1_H12(AAT-or-GAC)	b1_H12; b1_H6	612/3059/3853	0.040	0.0044	1.28E-18
b1_H9(AAT)/b1_OH9(*)	b1_H9; b1_OH9	346/2616/4572	0.036	0.0049	2.45E-12
b1_H7(AAC-or-AGC-or-GAC)/b1_OH7(*)	b1_OH7; b1_H7	350/2618/4566	0.035	0.0049	2.73E-12
b1_H7(AAC-or-AGC-or-GAC)/b1_H9(AAT)	b1_H9; b1_H7	346/2612/4566	0.036	0.0049	2.86E-12
b1_H6(AAC-or-AGC)/b1_H9(AAT)	b1_H9; b1_H6	346/2375/3853	0.037	0.0052	4.58E-12
b1_H1(AAC)/b1_H12(AAT-or-GAC)	b1_H12; b1_H1	612/1933/1498	0.039	0.0057	3.72E-11
b1_H12(AAT-or-GAC)/b1_H13(AGC)	b1_H13; b1_H12	564/1126/612	-0.050	0.0075	5.64E-11
b1_H13(AGC)/b1_OH13(*)	b1_H13; b1_OH13	564/2922/4048	-0.023	0.0047	6.72E-07

Associations of diplotypes with HDL-C levels (the top 10 results in terms of *P *value).

Effect sizes (mmol/l) shown are β-coefficients and measured as additive effects, which correspond to the average change in HDL-C levels when one major haplotype cluster is replaced with one minor haplotype cluster.

**Table 6:** Associations of diplotypes described in Figure 2.

Diplotypes	Minor; Major	Diplotype frequency	Effect	Standard error	*P *value
b1_H1(AAC)/b1_H9(AAT)	b1_H9; b1_H1	346/1503/1498	0.033	0.0065	7.13E-07
b1_H1(AAC)/b1_H15(GAC)	b1_H15; b1_H1	29/430/1498	0.035	0.012	0.0048
b1_H1(AAC)/b1_H13(AGC)	b1_H13; b1_H1	564/1791/1498	-0.0093	0.0057	0.13

Associations of diplotypes with HDL-C levels.

Effect sizes (mmol/l) shown are β-coefficients and measured as additive effects, which correspond to the average change in HDL-C levels when one major haplotype cluster is replaced with one minor haplotype cluster.

HL plays a key role in the interconversion between two HDL subspecies and the indirect pathway of hepatic cholesterol uptake in reverse cholesterol transport with involvement of HDL [13,14]. HDL-C levels are used clinically to evaluate the risk of developing cardiovascular disease [21]. The two SNPs (rs1800588 and rs2070895) in the promoter region are in almost perfect linkage disequilibrium (R^2 ^= 0.97) [24], and both the promoter SNP (rs1800588) and the intronic SNP (rs261332) show strong LD (R^2 ^= 0.92). The rs2070895 SNP showed a highly significant association, with a 0.057 mmol/l increase per A allele (*P *= 8 × 10^-10^) [24]. In the present study, the rs261332 showed a significant association (*P *= 3.03 × 10^-12^), with a 0.035 mmol/l increase per T allele. The rs11631342 also showed a significant association (*P *= 5.66 × 10^-6^), with a 0.037 mmol/l increase per G allele. The diplotypes b1_H12(AAT-or-GAC)/b1_OH12(*) (Table 5) showed a strong association (*P *= 9.09 × 10^-19^), with a 0.04 mmol/l increase per (AAT-or-GAC) haplotype cluster. Transporting cholesterol from peripheral tissues to the liver, HL interferes with the interconversions of the mature HDL particles and acts on the triglyceride-rich HDL, which is able to undergo hydrolysis to form small HDL particles, which are then transported to begin anew the process of cholesterol uptake [13]. Therefore, carriers of rs261332 T-allele and rs11631342 G-allele may have high HDL-C levels. The effect of these polymorphisms on HL synthesis may be an increased susceptibility to cardiovascular disease. The rs1800588 T-allele, rs2070895 A-allele, rs261332 T-allele, and rs11631342 G-allele may be of clinical relevance, and conferring protection against cardiovascular disease, as there is increasing evidence from population studies that increasing HDL-C levels reduces the risk of cardiovascular disease [17-20]. This finding requires replication in an independent population sample.

## Conclusions

Significant haplotype structure has been routinely investigated to identify haplotypes carrying causative mutations. Many software tools like Haploview [2] and PLINK [3] provide statistical methods for haplotype association tests, most of which focus on only a single-haplotype. Notably, Durrant et al. showed through the simulation studies and empirical data that their association analysis based on haplotype clusters had increased power over single-locus or single-haplotype tests [10]. That is, they demonstrated the superiority of haplotype cluster-based association analysis, which extended the previous method based on the single-haplotype. Nevertheless, the haplotype cluster-based analysis can have the more limitation of association analysis compared with diplotype-based analysis as if allele-based analysis can have less diversity than genotype-based analysis. Meanwhile, Luo et al. demonstrated that DTR analysis based on diplotypes outperformed other, more common association methods [11]; however, they did not apply haplotype clusters to the DTR analysis.

We developed a novel method that can yield a synergistic effect by combining the positive aspects of analyses based on haplotype clusters and diplotypes. The Diplotyper software employs Haploview tool, utilizing LD block and haplotypes with a frequency threshold. The software also uses PLINK tool to generate all possible haplotype pairs in given genotypes of samples and perform association analysis using linear or logistic regression. In addition to employing these existing software tools, we designed a new procedure to cluster haplotypes of interest from a large set of haplotypes and implemented a function to generate the patterns of all possible diplotypes from these haplotype clusters. Finally, a function to transform diplotypes into PLINK formats was implemented. All of these processes were fully automated. We tested our method by conducting an association analysis between *LIPC *SNPs or diplotypes and HDL-C levels. The result showed that our approach can identify more precise and distinct signals compared with single-locus tests.

## List of abbreviations used

HDL: high-density lipoprotein; HDL-C: high-density lipoprotein cholesterol; LD: linkage disequilibrium; EM: Expectation-Maximization; SNP: single nucleotide polymorphism; HLA: human leukocyte antigen; ART: assisted reproduction treatments; DTR: diplotype trend regression; HL: hepatic lipase; CEU: Caucasians of European descent from Utah; KARE: Korea Association Resource; KoGES: Korean Genome Epidemiology Study; KNIH: Korean National Institute of Health; MAF: minor allele frequency; HWE: Hardy-Weinberg equilibrium; BMI: body mass index.

## Competing interests

The authors declare that they have no competing interests.

## Authors' contributions

SK designed the algorithm and implemented it for the Diplotyper, and drafted the manuscript. KK conceived of the haplotype cluster-based analysis and helped to draft the manuscript. IK carried out the statistical and physiological analyses. KP and JK participated in the design of the study and the statistical analysis. CS and NC performed data acquisition.

## Acknowledgements

This study was supported by a grant from the Korea Health technology R&D Project, Ministry of Health & Welfare, Republic of Korea (A110749), grants from Korea Centers for Disease Control and Prevention (4845-301, 4851-302, 4851-307), and the Priority Centers Program of the National Research Foundation of Korea (NRF), which is funded by the Ministry of Education, Science, and Technology (No. 2009-0093821).

## Declarations

The publication costs for this article were funded by the first author.

This article has been published as part of *BMC Medical Genomics *Volume 6 Supplement 2, 2013: Selected articles from the Second Annual Translational Bioinformatics Conference (TBC 2012). The full contents of the supplement are available online at http://www.biomedcentral.com/bmcmedgenomics/supplements/6/S2.
